# Outcomes and Predictors of Mortality in Perforated Versus Non-Perforated Peptic Ulcer Disease: A U.S. Nationwide Propensity-Matched Analysis, 2016–2021

**DOI:** 10.3390/jcm15114358

**Published:** 2026-06-04

**Authors:** Abdullah Sultany, Adishwar Rao, Amlish Gondal, Abhinay Theli, Rahul Chikatimalla, Mohamed A. Omar, Rewanth Katamreddy, Raja Chandra Chakinala, Mohammad Sulaiman Sultany, Dushyant Singh Dahiya, Subash Ghimire, Michael J. Georgetson

**Affiliations:** 1 Department of Internal Medicine, Guthrie Robert Packer Hospital, 1 Guthrie Square, Sayre, PA 18840, USA; 2 Department of Gastroenterology and Hepatology, Guthrie Robert Packer Hospital, Sayre, PA 18840, USArewanth.katamreddy@guthrie.org (R.K.);; 3 Department of Internal Medicine, University of Texas Health Science Center, Tyler, TX 75708, USA; 4 Department of Gastroenterology, University of Kansas, Wichita, KS 67214, USA; 5 Department of General and Bariatric Surgery, Guthrie Robert Packer Hospital, Sayre, PA 18840, USA; 6 Division of Gastroenterology, Hepatology & Motility, School of Medicine, University of Kansas, Kansas City, KS 66160, USA

**Keywords:** peptic ulcer disease, perforated peptic ulcer, mortality, sepsis, acute kidney injury, healthcare costs, National Inpatient Sample, COVID-19, risk stratification, emergency surgery

## Abstract

**Background/Objectives:** Perforated peptic ulcer (PPU) represents a surgical emergency with substantial morbidity and mortality. Despite declining overall peptic ulcer disease (PUD) incidence, contemporary population-based data comparing outcomes between perforated and non-perforated PUD remain limited, particularly during the COVID-19 pandemic. This study aimed to characterize the clinical and economic burden of PPU and identify independent predictors of adverse outcomes. **Methods:** We conducted a retrospective cohort study using the National Inpatient Sample (2016–2021), identifying 2,561,379 weighted hospitalizations for PUD. Hospitalizations were stratified by the presence (*n* = 207,970, 8.1%) or absence (*n* = 2,353,409, 91.9%) of perforation. The primary outcome was in-hospital mortality; secondary outcomes included sepsis, septic shock, acute kidney injury (AKI), prolonged length of stay (≥7 days), and high healthcare costs (≥$12,000). We performed 1:1 propensity score matching and multivariable logistic regression to assess independent predictors of adverse outcomes in the PPU cohort. **Results:** After propensity matching, PPU demonstrated significantly higher mortality than non-perforated PUD (7.2% vs. 3.0%, *p* < 0.001), along with increased rates of sepsis (21.8% vs. 8.2%, *p* < 0.001), septic shock (12.9% vs. 3.5%, *p* < 0.001), and AKI (29.5% vs. 22.8%, *p* < 0.001). Nearly half (45.6%) of PPU admissions exceeded 7 days, and 69.2% incurred costs ≥$12,000. Among PPU hospitalizations, multivariable analysis identified age ≥ 65 years (aOR 5.79, 95% CI 4.60–7.28), liver cirrhosis (aOR 1.93, 95% CI 1.62–2.30), chronic heart failure (aOR 1.73, 95% CI 1.56–1.92), and concurrent COVID-19 infection (aOR 4.35, 95% CI 3.46–5.47) as independent predictors of mortality. Chronic kidney disease strongly predicted AKI (aOR 2.81, 95% CI 2.64–2.98). Mortality increased from 6.9% (2016) to 8.0% (2021) in PPU hospitalizations (*p* < 0.001 for trend), with higher rates observed during the 2020 to 2021 period that coincided with the COVID-19 pandemic. **Conclusions:** Perforation complicates approximately 8% of hospitalized PUD cases but accounts for disproportionate mortality, sepsis, organ failure, and healthcare costs. Older age, cirrhosis, heart failure, and chronic kidney disease identify high-risk PPU patients requiring intensive monitoring and aggressive management. These findings support risk-stratified approaches focused on timely diagnosis, source control, and sepsis management to reduce the clinical and economic burden of PPU.

## 1. Introduction

Peptic ulcer disease (PUD) remains a significant clinical and economic burden in the United States. This persists despite a declining incidence due to *Helicobacter pylori* eradication and proton pump inhibitor therapy [1,2]. Among its complications, a perforated peptic ulcer (PPU) is a surgical emergency. It carries considerable morbidity, mortality, and healthcare resource use, with reported case-fatality rates from 5% to 15% or higher [3,4]. Yet there is a paucity of contemporary U.S. population-based data directly comparing outcomes between PUD admissions with and without perforation.

Several patient- and system-level factors influence outcomes in perforated PUD. Notably, advanced age, cardiovascular disease, cirrhosis, and exposure to nonsteroidal anti-inflammatory drugs or antithrombotic agents are established risk factors for ulcer complications [5,6]. The COVID-19 pandemic further complicated this landscape by introducing additional challenges to time-sensitive surgical care, including delayed presentation and strained hospital resources; however, its impact on national PPU outcomes remains unclear [7,8].

Sepsis is a critical determinant of outcomes in gastrointestinal surgical emergencies and is a frequent complication of PPU. In the United States, the incidence, mortality, and costs associated with sepsis have increased, particularly among older adults. From 2019 to 2021, sepsis-related in-hospital mortality among adults aged 65 or older increased by 37.5% [9]. Abdominal sepsis related to PPU has reported mortality rates of 18% to 20%; however, previous studies have rarely isolated the risk specific to perforation [10].

Existing analyses of the National Inpatient Sample (NIS) have often grouped perforation with other complications of peptic ulcer disease, such as hemorrhage, which limits accurate estimation of outcomes attributable specifically to perforation [11]. Few contemporary studies have directly compared perforated and non-perforated PUD within a single nationally representative cohort, and the adjusted associations between perforation and sepsis, septic shock, acute kidney injury (AKI), length of stay, hospital costs, and pandemic-era mortality trends have not been systematically examined. By isolating perforation without concurrent hemorrhage and applying both propensity score matching and multivariable adjustment that account for patient and hospital-level characteristics, the present study addresses these gaps and provides contemporary, perforation-specific estimates spanning the pre-pandemic and COVID-19 periods.

This study uses NIS data from 2016 to 2021 to compare in-hospital outcomes and resource use between PUD admissions with and without perforation. The objectives are to: (1) estimate the adjusted odds of mortality, sepsis or septic shock, AKI, prolonged LOS, and high hospital charges and costs associated with perforation; (2) identify predictors of adverse outcomes among PPU patients; and (3) assess trends in these outcomes before (2016–2019) and during (2020–2021) the COVID-19 pandemic. We hypothesize that: (i) PPU is independently associated with increased odds of mortality and major complications; (ii) among PPU patients, older age, cirrhosis, heart failure, and concurrent COVID-19 infection predict adverse outcomes; and (iii) clinical and economic outcomes worsened during the COVID-19 era compared with the pre-pandemic baseline.

## 2. Materials and Methods

### 2.1. Data Source and Cohort Selection

We queried the NIS database provided by the Agency for Healthcare Research and Quality (AHRQ) under the Healthcare Cost and Utilization Project (HCUP) to perform a retrospective observational study [12]. The NIS is the largest all-payer inpatient database publicly available for outcomes research in the United States, comprising a stratified 20% sample of discharges from all participating community hospitals, from which national estimates are derived using survey weights. We identified all hospitalizations for peptic ulcer disease during the study period (2016–2021) using International Classification of Diseases, Tenth Revision, Clinical Modification (ICD-10-CM) codes (Supplementary Table S1). We excluded encounters for patients under 18 years of age and those with missing data on age, sex, or in-hospital mortality. The cohort was then stratified into two groups based on the presence or absence of perforation.

To isolate perforation as the exposure of interest, we defined the perforated group using ICD-10-CM codes denoting peptic ulcer with perforation but without concurrent hemorrhage. Codes denoting combined hemorrhage and perforation were not included, in order to avoid misattribution of bleeding-related outcomes to perforation and to define a clinically homogeneous perforation cohort. The complete code set used to define the overall cohort, the perforation subgroup, comorbidities, and outcomes is provided in Supplementary Table S1.

### 2.2. Study Variables and Outcomes

We examined demographic characteristics, hospital-level characteristics, comorbidities, and clinical and economic outcomes. Hospital-level characteristics included hospital region, location and teaching status, bed size, and primary expected payer. Total hospital charges were adjusted for annual inflation using the Consumer Price Index. Comorbidities comprised cardiovascular and cerebrovascular conditions (hyperlipidemia, hypertension, heart failure, prior myocardial infarction, prior percutaneous coronary intervention, prior coronary artery bypass grafting, prior stroke, and smoking/tobacco use), endocrine and metabolic conditions (diabetes mellitus, obesity, hypothyroidism), renal disease (chronic kidney disease/end-stage renal disease), pulmonary conditions (chronic obstructive pulmonary disease, obstructive sleep apnea, and COVID-19), liver disease (alcoholic liver disease, toxic liver disease, and liver cirrhosis/fibrosis), and nutritional anemia.

The primary outcome was in-hospital mortality. Secondary outcomes included sepsis, septic shock, acute kidney injury (AKI), other/unspecified shock, prolonged length of stay (≥7 days), and significant economic burden, defined using prespecified pragmatic analytic thresholds (rather than externally validated clinical cutoffs) of total charges ≥ $40,000 and total costs ≥ $12,000. Total charges and costs were dichotomized at these prespecified thresholds as pragmatic markers of high-resource hospitalizations. Because hospital charges and costs are highly right-skewed, dichotomization provides a clinically interpretable basis for comparison between groups.

### 2.3. Statistical Analysis

We assessed the distribution of continuous variables using histograms and quantile plots. Categorical data were summarized as weighted counts and percentages, and continuous data as means. Categorical variables were compared using the Pearson chi-square test and continuous variables using the *t* test. All analyses incorporated the discharge weight (DISCWT), hospital cluster, and stratum variables provided by HCUP to generate nationally representative estimates that account for the complex survey design. The NIS contains minimal missingness for the primary outcome and core analytic variables; encounters with missing data on age, sex, or mortality were excluded as described above, and no imputation was performed.

To minimize confounding from baseline differences between perforated and non-perforated groups, we estimated propensity scores using a logistic regression model with perforation status as the dependent variable. The propensity score model included all demographic characteristics, comorbidities, and hospital-level characteristics (region, location/teaching status, bed size, and primary payer); the complete covariate list is provided in Supplementary Table S2. We performed 1:1 nearest-neighbor matching without replacement using the *psmatch2* command in Stata, applying a caliper of 0.1 of the propensity score and restricting matches to the region of common support. These specifications yielded 40,364 matched pairs. The relatively conservative caliper and common-support restriction, rather than a limited donor pool, account for the proportion of perforated encounters not retained in the matched cohort; matching without replacement was feasible given the large donor pool of non-perforated cases (*n* = 2,353,409) relative to perforated cases (*n* = 207,970). Covariate balance before and after matching was assessed using the standardized percent bias for each covariate and overall summary measures (mean and median absolute bias and Rubin’s B), with standardized differences below 10% considered indicative of adequate balance (Supplementary Table S3 and Supplementary Figure S1).

Within the perforated PUD cohort, we performed multivariable logistic regression for each clinical outcome to identify independent predictors, reporting adjusted odds ratios (aOR) with 95% confidence intervals (CI). To verify the stability of these models, we examined multicollinearity among predictors using variance inflation factors (VIF); all values were well below conventional thresholds of concern (Supplementary Table S4). Temporal trends in in-hospital mortality across the study period were evaluated using the Cochran–Armitage test for trend. Statistical significance was defined as a two-sided *p*-value < 0.05. All analyses were performed using Stata version 18 (StataCorp LLC, College Station, TX, USA).

### 2.4. Institutional Review Board Approval

Institutional Review Board review was not required because the study used publicly available, fully de-identified data. In accordance with HCUP data use policies, cells representing fewer than 11 observations were not reported.

## 3. Results

### 3.1. Demographics and Comorbidity Burden

We analyzed 2,561,379 weighted hospitalizations for peptic ulcer disease, of which 207,970 (8.1%) involved perforation and 2,353,409 (91.9%) did not. Hospitalizations with perforation were, on average, slightly younger (mean age 63.9 vs. 65.7 years, *p* < 0.001), more often female (51.7% vs. 49.2%, *p* < 0.001), and differed significantly by race/ethnicity (White: 71.3% vs. 68.5%; Black: 13.2% vs. 14.5%; Hispanic: 8.4% vs. 9.9%; *p* < 0.001).

**Figure 1:**
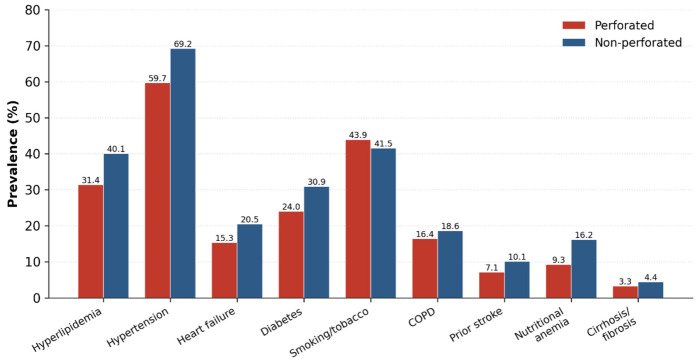
Comorbidity prevalence in perforated versus non-perforated peptic ulcer disease.

Compared with non-perforated hospitalizations, those with perforation had lower proportions of several cardiometabolic comorbidities, including hyperlipidemia (31.4% vs. 40.1%, *p* < 0.001), hypertension (59.7% vs. 69.2%, *p* < 0.001), chronic heart failure (15.3% vs. 20.5%, *p* < 0.001), and diabetes mellitus (24.0% vs. 30.9%, *p* < 0.001). Conversely, smoking/tobacco use was more prevalent in the perforation group (43.9% vs. 41.5%, *p* < 0.001) (Figure 1; Table 1).

### 3.2. Hospital Metrics

Medicare was the predominant payer across the cohort, although hospitalizations with perforation had relatively lower Medicare coverage (53.8% vs. 60.0%, *p* < 0.001) and higher proportions of private insurance (22.3% vs. 19.4%, *p* < 0.001) and self-pay (6.0% vs. 4.2%, *p* < 0.001). Hospitalizations with perforation incurred substantially higher inflation-adjusted total charges (mean $137,815 vs. $90,390) and total costs (mean $32,245 vs. $20,710), and a longer mean length of stay (9.3 vs. 6.6 days; all *p* < 0.001).

### 3.3. Outcomes

In crude analyses, hospitalizations with perforation had significantly higher rates of adverse outcomes than those without perforation. In-hospital mortality was more than double (7.2% vs. 3.3%, *p* < 0.001), with higher rates of sepsis (21.8% vs. 8.5%, *p* < 0.001), septic shock (12.9% vs. 3.6%, *p* < 0.001), and AKI (29.5% vs. 26.0%, *p* < 0.001). The perforation group also had a greater proportion of prolonged hospitalizations (length of stay ≥ 7 days: 45.6% vs. 29.5%, *p* < 0.001) and high-cost encounters (total cost ≥ $12,000: 69.2% vs. 48.0%, *p* < 0.001).

**Figure 2:**
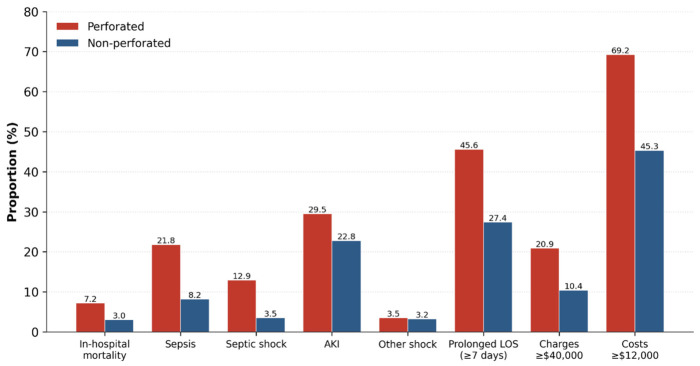
Propensity-matched clinical and economic outcomes (*n* = 40,364 pairs).

After 1:1 propensity score matching, 40,364 hospitalizations were retained in each group, with excellent covariate balance (all standardized differences < 4%; Supplementary Table S3). Matched comparisons confirmed the crude findings, with the perforation group showing persistently higher rates of in-hospital mortality (7.2% vs. 3.0%, *p* < 0.001), sepsis (21.8% vs. 8.2%, *p* < 0.001), septic shock (12.9% vs. 3.5%, *p* < 0.001), AKI (29.5% vs. 22.8%, *p* < 0.001), other/unspecified shock (3.5% vs. 3.2%, *p* < 0.001), prolonged length of stay (45.6% vs. 27.4%, *p* < 0.001), high total charges (≥$40,000: 20.9% vs. 10.4%, *p* < 0.001), and high total costs (≥$12,000: 69.2% vs. 45.3%, *p* < 0.001) (Figure 2; Table 2).

In multivariable logistic regression within the perforated PUD cohort, increasing age was the strongest predictor of in-hospital mortality (45–64 years: aOR 2.80, 95% CI 2.26–3.48; ≥65 years: aOR 5.79, 95% CI 4.60–7.28) relative to patients aged 18–44 years. Liver cirrhosis/fibrosis (aOR 1.93, 95% CI 1.62–2.30), chronic heart failure (aOR 1.73, 95% CI 1.56–1.92), and COPD (aOR 1.55, 95% CI 1.40–1.72) were each independently associated with higher mortality. Concurrent COVID-19 infection was associated with a more than fourfold increase in the odds of in-hospital death (aOR 4.35, 95% CI 3.46–5.47) and with significantly higher odds of sepsis, septic shock, AKI, and other/unspecified shock (all *p* < 0.001). Chronic kidney disease was the strongest predictor of AKI (aOR 2.81, 95% CI 2.64–2.98). Female sex was associated with modestly reduced odds across all adverse outcomes (all *p* < 0.05). Full regression results for all outcomes are presented in Table 3 and Supplementary Table S5.

Several conditions, including smoking/tobacco use (mortality aOR 0.57, 95% CI 0.52–0.62), hypertension (aOR 0.77, 95% CI 0.70–0.84), and hyperlipidemia (aOR 0.65, 95% CI 0.59–0.72), showed paradoxical inverse associations with mortality. Formal collinearity diagnostics did not support multicollinearity as an explanation, as all variance inflation factors were well below conventional thresholds (mean VIF 1.23; Supplementary Table S4); these associations are interpreted in the context of residual confounding and survivorship bias in the Discussion.

### 3.4. Mortality Trends

**Figure 3:**
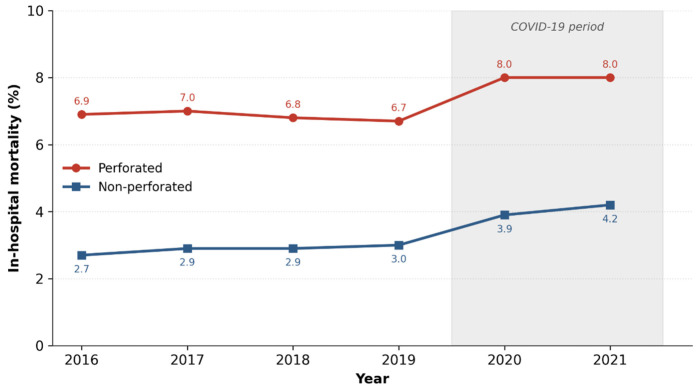
In-hospital mortality trends, 2016 to 2021.

In-hospital mortality rose across the study period in both groups. Among hospitalizations with perforation, mortality increased from 6.7% in 2019 to 8.0% in 2020 and 2021, with an overall upward trend from 6.9% in 2016 to 8.0% in 2021 (*p* < 0.001 for trend). Mortality in non-perforated PUD similarly increased from 2.7% in 2016 to 4.2% in 2021 (*p* < 0.001 for trend). The increases in both groups were greater during the 2020 to 2021 period, which coincided with the COVID-19 pandemic (Figure 3; Table 4).

## 4. Discussion

This nationwide analysis of more than 2.5 million peptic ulcer disease (PUD) hospitalizations demonstrates that perforation, although complicating only 8% of admissions, carries a disproportionate burden of mortality, organ failure, and resource use. After rigorous propensity score matching that balanced demographic, comorbidity, and hospital-level characteristics, hospitalizations with perforated peptic ulcer (PPU) had more than double the in-hospital mortality of non-perforated PUD (7.2% vs. 3.0%), together with markedly higher rates of sepsis, septic shock, acute kidney injury (AKI), prolonged hospitalization, and high-cost care. Within the perforated cohort, advanced age, liver cirrhosis, chronic heart failure, chronic kidney disease, COPD, and concurrent COVID-19 infection independently predicted adverse outcomes.

These mortality and morbidity estimates align with the contemporary literature. Reported short-term mortality after PPU ranges widely, with population-based and pooled estimates of 30-day mortality reaching approximately 23.5%, and our in-hospital figure of 7.2% falls within the lower end of this range, consistent with the shorter observation window inherent to inpatient administrative data [13,14,15]. The persistent burden of perforation despite declining overall PUD incidence echoes global epidemiologic data showing that, although age-standardized PUD rates have fallen over recent decades, the decline has plateaued and complications continue to affect an aging population exposed to nonsteroidal anti-inflammatory drugs, antithrombotic agents, and *Helicobacter pylori* [15,16]. A recent global multicenter study of surgery for PPU similarly reported substantial 30-day morbidity and mortality with a median hospital stay comparable to that observed here [17]. Notably, patients with perforation in our cohort were younger and carried fewer documented cardiometabolic comorbidities than those without perforation, yet experienced substantially worse outcomes. This suggests that perforation is strongly associated with the excess risk, independent of baseline comorbidity burden, rather than the excess risk being explained by comorbidity alone.

Our study extends prior National Inpatient Sample analyses, which have frequently grouped perforation with other PUD complications and thereby obscured perforation-specific risk [11]. By isolating perforation without concurrent hemorrhage and applying both propensity matching and multivariable modeling adjusted for hospital-level factors, we provide contemporary, adjusted estimates of the independent association between perforation and a comprehensive set of clinical and economic outcomes, including the pandemic-era period.

Age was the dominant determinant of mortality, with patients aged 65 years and older having nearly sixfold higher adjusted odds of death than those aged 18–44 years. This is consistent with established perforated-ulcer risk scores, including the Boey and Peptic Ulcer Perforation (PULP) scores, which assign substantial prognostic weight to advanced age [18,19,20]. Among comorbidities, liver cirrhosis nearly doubled the odds of in-hospital death. This finding is biologically coherent: cirrhosis impairs surgical and infection outcomes through coagulopathy, adaptive immune dysfunction, cirrhotic cardiomyopathy, and impaired hemodynamic reserve, and is a recognized driver of poor outcomes after non-hepatic abdominal surgery [21]. Chronic heart failure and COPD likewise emerged as independent predictors, in keeping with their established roles in perioperative physiologic intolerance, and chronic kidney disease was the strongest predictor of AKI, reflecting diminished renal reserve in the setting of sepsis and hemodynamic stress.

The burden of sepsis and organ failure was substantial and represents the principal pathway to mortality in this cohort. More than one in five matched perforation hospitalizations developed sepsis and nearly 13% developed septic shock, rates far exceeding those in non-perforated disease. Perforation produces peritoneal contamination and intra-abdominal infection, which remains among the most lethal sources of sepsis, with reported hospital mortality of 23% to 38% and outcomes critically dependent on timely source control and appropriate antimicrobial therapy [22]. These mechanisms are consistent with prior PPU cohorts and emergency-laparotomy data identifying sepsis and multiorgan failure as the dominant contributors to postoperative death [17,23]. The high rate of AKI we observed, strongly predicted by baseline chronic kidney disease, is similarly congruent with sepsis-associated renal injury as a marker of physiologic decompensation.

The rising mortality we observed parallels recent national data on increasing peptic ulcer disease-related mortality in the United States [24]. These temporal findings warrant cautious interpretation. Mortality rose in both groups, with the steepest increases during the 2020 to 2021 pandemic period, and concurrent COVID-19 infection was associated with a more than fourfold increase in the adjusted odds of death, although our design cannot establish causation. During the pandemic, emergency surgical services faced delayed presentations and reduced operative volumes, and surgical mortality rose irrespective of confirmed COVID-19 status [25]. Delayed presentation may lead to greater peritoneal contamination and more advanced sepsis, and COVID-19 itself can compound this insult through endothelial dysfunction, hypercoagulability, and dysregulated inflammation, particularly in older adults [26]. The parallel rise among non-perforated hospitalizations points to a system-wide effect rather than one specific to perforation, and administrative data cannot distinguish death caused by COVID-19 from death merely occurring with incidental infection. Prior institutional analyses of peptic ulcer perforation during the pandemic similarly reported altered management and outcomes [27,28], although emergency surgical care was largely maintained. These temporal associations should be regarded as hypothesis-generating.

Several comorbidities, including smoking, hypertension, hyperlipidemia, prior percutaneous coronary intervention, and nutritional anemia, showed paradoxical inverse associations with mortality. These are unlikely to reflect multicollinearity, as all variance inflation factors were well below conventional thresholds (mean 1.23; Supplementary Table S4). Instead, they most likely reflect residual confounding and selection effects inherent to a disease-restricted cohort. Conditioning on an index event such as perforation can make established risk factors appear spuriously protective through collider (index-event) bias, as described for the apparent protective effect of smoking in such samples [29]. Survivorship bias may also contribute, as the frailest patients with heavy comorbidity burden may not survive to present with perforation. These associations should not be interpreted as causal or protective.

This study has notable strengths, including a large, nationally representative sample, adherence to recommended NIS methodological standards, and the combined use of propensity score matching incorporating hospital-level covariates and multivariable regression [30]. Several limitations should be acknowledged. As an administrative dataset, the NIS is subject to coding misclassification and lacks granular clinical detail. Unmeasured factors include ulcer size and location, symptom-to-surgery time, hemodynamic and laboratory values, operative approach, and post-discharge outcomes, all of which influence prognosis and are incorporated into validated risk scores [18,31,32]. The database captures hospitalizations rather than individual patients, precluding longitudinal follow-up and assessment of readmission or recurrence. Although our propensity model incorporated hospital-level characteristics, residual confounding from unmeasured factors such as frailty, nutritional status, medication exposures, and delays in presentation cannot be excluded. Finally, total charges and costs are derived constructs subject to inflation and institutional variation, and were dichotomized as pragmatic markers of high-resource care.

## 5. Conclusions

In this nationwide cohort, perforation complicated approximately 8% of PUD hospitalizations but accounted for a disproportionate burden of mortality, sepsis, organ failure, prolonged hospitalization, and cost, with associations that persisted after propensity matching and multivariable adjustment. Advanced age, liver cirrhosis, chronic heart failure, chronic kidney disease, COPD, and concurrent COVID-19 infection identified patients at particularly high risk. The rise in mortality observed during the 2020 to 2021 period, which cannot be attributed to COVID-19 on the basis of this descriptive analysis, may reflect the vulnerability of time-sensitive emergency surgical care to systemic disruption. Collectively, these findings support early recognition, prompt source control, aggressive sepsis management, and risk-stratified care for patients with perforated peptic ulcers, and they suggest that maintaining resilient emergency surgical pathways may be important during periods of health-system strain.

## Figures and Tables

**Table 1:** Baseline characteristics of hospitalizations with peptic ulcer disease, stratified by perforation status (weighted).

Characteristic	Perforation (*n* = 207,970)	No Perforation (*n* = 2,353,409)	*p*-Value
Age (years), mean	63.9	65.7	<0.001
Age group			<0.001
18–44	26,710 (12.8)	252,300 (10.7)	
45–64	73,760 (35.5)	762,545 (32.4)	
≥65	107,500 (51.7)	1,338,564 (56.9)	
Female sex	107,540 (51.7)	1,157,900 (49.2)	<0.001
Race/ethnicity			<0.001
White	144,015 (71.3)	1,568,384 (68.5)	
Black	26,755 (13.2)	332,170 (14.5)	
Hispanic	16,895 (8.4)	227,190 (9.9)	
Asian/Pacific Islander	7585 (3.8)	84,000 (3.7)	
Native American	1475 (0.7)	14,660 (0.6)	
Other	5340 (2.6)	62,810 (2.7)	
Primary payer			<0.001
Medicare	111,650 (53.8)	1,410,574 (60.0)	
Medicaid	30,550 (14.7)	319,535 (13.6)	
Private	46,320 (22.3)	455,350 (19.4)	
Self-pay	12,435 (6.0)	98,020 (4.2)	
No charge	1015 (0.5)	10,075 (0.4)	
Other	5735 (2.8)	56,835 (2.4)	
Hospital region			<0.001
Northeast	33,500 (16.1)	400,935 (17.0)	
Midwest	45,815 (22.0)	537,240 (22.8)	
South	83,610 (40.2)	930,825 (39.6)	
West	45,045 (21.7)	484,409 (20.6)	
Location/teaching status			<0.001
Rural	19,250 (9.3)	180,495 (7.7)	
Urban non-teaching	45,500 (21.9)	512,060 (21.8)	
Urban teaching	143,220 (68.9)	1,660,855 (70.6)	
Hospital bed size			0.030
Small	43,690 (21.0)	482,644 (20.5)	
Medium	62,225 (29.9)	698,024 (29.7)	
Large	102,055 (49.1)	1,172,740 (49.8)	
Comorbidities			
Hyperlipidemia	65,215 (31.4)	944,005 (40.1)	<0.001
Hypertension	124,165 (59.7)	1,629,494 (69.2)	<0.001
Chronic heart failure	31,750 (15.3)	483,250 (20.5)	<0.001
Prior myocardial infarction	9490 (4.6)	164,655 (7.0)	<0.001
Prior PCI	9420 (4.5)	157,545 (6.7)	<0.001
Prior CABG	415 (0.2)	6915 (0.3)	<0.001
Obesity	33,055 (15.9)	385,425 (16.4)	0.012
Smoking/tobacco use	91,340 (43.9)	976,385 (41.5)	<0.001
COPD	34,090 (16.4)	438,895 (18.6)	<0.001
Obstructive sleep apnea	11,265 (5.4)	163,290 (6.9)	<0.001
Prior stroke	14,675 (7.1)	238,495 (10.1)	<0.001
Alcoholic liver disease	725 (0.3)	11,325 (0.5)	<0.001
Liver cirrhosis/fibrosis	6930 (3.3)	102,930 (4.4)	<0.001
Toxic liver disease	35 (0.0)	445 (0.0)	0.764
Diabetes mellitus	49,835 (24.0)	727,360 (30.9)	<0.001
Hypothyroidism	23,420 (11.3)	312,295 (13.3)	<0.001
Nutritional anemia	19,290 (9.3)	380,875 (16.2)	<0.001
COVID-19	2410 (1.2)	26,770 (1.1)	0.696

Data are presented as *n* (%) unless otherwise indicated. Percentages are weighted. PCI = percutaneous coronary intervention; CABG = coronary artery bypass grafting; COPD = chronic obstructive pulmonary disease. *p*-values from Pearson chi-square (categorical) or *t* test (continuous).

**Table 2:** Crude and propensity-matched clinical and economic outcomes, perforated versus non-perforated peptic ulcer disease.

Outcome	Crude—Perf (*n* = 207,970)	Crude—No Perf (*n* = 2,353,409)	*p*	Matched—Perf (*n* = 40,364)	Matched—No Perf (*n* = 40,364)	*p*
In-hospital mortality	15,020 (7.2)	76,505 (3.3)	<0.001	2900 (7.2)	1212 (3.0)	<0.001
Sepsis	45,320 (21.8)	200,500 (8.5)	<0.001	8816 (21.8)	3303 (8.2)	<0.001
Septic shock	26,780 (12.9)	84,965 (3.6)	<0.001	5191 (12.9)	1401 (3.5)	<0.001
Acute kidney injury	61,320 (29.5)	612,685 (26.0)	<0.001	11,902 (29.5)	9184 (22.8)	<0.001
Other/unspecified shock	7210 (3.5)	83,725 (3.6)	0.344	1401 (3.5)	1281 (3.2)	<0.001
Prolonged LOS (≥7 days)	94,770 (45.6)	694,625 (29.5)	<0.001	18,394 (45.6)	11,038 (27.4)	<0.001
Total charges ≥ $40,000	43,830 (21.1)	263,385 (11.2)	<0.001	8458 (20.9)	4180 (10.4)	<0.001
Total cost ≥ $12,000	143,910 (69.2)	1,128,720 (48.0)	<0.001	27,910 (69.2)	18,298 (45.3)	<0.001

Data are *n* (%), weighted. Matched cohort derived from 1:1 nearest-neighbor propensity score matching (caliper 0.1, common support). LOS = length of stay.

**Table 3:** Independent predictors of in-hospital mortality and sepsis among hospitalizations with perforated peptic ulcer disease (multivariable logistic regression).

Predictor	In-Hospital Mortality aOR (95% CI)	Sepsis aOR (95% CI)
Age group		
45–64	2.80 (2.26–3.48)	1.52 (1.39–1.66)
≥65	5.79 (4.60–7.28)	1.73 (1.57–1.91)
Female sex	0.91 (0.84–0.99)	0.92 (0.87–0.96)
Heart failure	1.73 (1.56–1.92)	1.51 (1.41–1.62)
Chronic kidney disease	1.41 (1.27–1.56)	1.14 (1.06–1.22)
Liver cirrhosis/fibrosis	1.93 (1.62–2.30)	1.30 (1.14–1.48)
COPD	1.55 (1.40–1.72)	1.53 (1.43–1.63)
Diabetes mellitus	1.04 (0.95–1.15)	1.13 (1.07–1.21)
Obesity	0.99 (0.89–1.11)	1.28 (1.19–1.37)
COVID-19	4.35 (3.46–5.47)	2.35 (1.93–2.86)
Hyperlipidemia	0.65 (0.59–0.72)	0.73 (0.69–0.77)
Hypertension	0.77 (0.70–0.84)	0.88 (0.83–0.93)
Smoking/tobacco use	0.57 (0.52–0.62)	0.74 (0.70–0.78)
Prior PCI	0.54 (0.42–0.69)	0.49 (0.42–0.57)
Nutritional anemia	0.67 (0.57–0.78)	0.74 (0.68–0.82)

aOR = adjusted odds ratio; CI = confidence interval. Models adjusted for demographics, comorbidities, and hospital-level characteristics (region, teaching status, bed size, primary payer). Full results for all outcomes, including septic shock, acute kidney injury, and other/unspecified shock, are provided in Supplementary Table S5. Inverse associations observed for some comorbidities (for example, smoking, hyperlipidemia, and hypertension) should not be interpreted as causal or protective; they most likely reflect residual confounding and selection effects, including index-event and survivorship bias, in this disease-restricted cohort.

**Table 4:** In-hospital mortality trends by year, 2016–2021 (%).

Group	2016	2017	2018	2019	2020	2021	*p*-Trend
Perforated	6.9	7.0	6.8	6.7	8.0	8.0	<0.001
Non-perforated	2.7	2.9	2.9	3.0	3.9	4.2	<0.001

Values are weighted in-hospital mortality (%). *p*-trend from Cochran–Armitage test.

## Data Availability

The data presented in this study are available from the Healthcare Cost and Utilization Project (HCUP) National Inpatient Sample (NIS) at https://www.hcup-us.ahrq.gov/nisoverview.jsp (accessed on 5 April 2026). These data were derived from a resource available in the public domain: HCUP National Inpatient Sample (NIS), Agency for Healthcare Research and Quality (AHRQ). Access to the NIS requires completion of the HCUP Data Use Agreement training.

## Supplementary Materials

The following supporting information can be downloaded at: https://www.mdpi.com/article/10.3390/jcm15114358/s1, Table S1: ICD-10-CM diagnosis codes used to define the peptic ulcer disease cohort, the perforated subgroup, comorbidities, and outcomes; Table S2: Covariates included in the propensity score model and the multivariable regression models; Table S3: Standardized percent bias for all covariates before and after 1:1 propensity score matching, with overall summary measures (mean and median absolute bias and Rubin’s B); Table S4: Variance inflation factors for the multivariable logistic regression models; Table S5: Complete multivariable logistic regression results for all five outcomes (in-hospital mortality, sepsis, septic shock, acute kidney injury, and other/unspecified shock); Figure S1: Propensity score distributions in the perforated and non-perforated groups before and after 1:1 nearest-neighbor matching.
