# Supplementary material for: Outcomes and Predictors of Mortality in Perforated Versus Non-Perforated Peptic Ulcer Disease: A U.S. Nationwide Propensity-Matched Analysis, 2016–2021
Source: J Clin Med. 2026 Jun 4;15(11):4358. doi: 10.3390/jcm15114358 (PMC13257457; doi:10.3390/jcm15114358)
Supplement: Supplementary file 1 [file jcm-15-04358-s001.zip › Supplemental Tables S3 S4 S5.pdf]

Supplemental Table S3. Standardized percent bias for all covariates before and after 1:1 propensity score matching, with overall summary measures (mean and median absolute bias and Rubin's B).

Covariate	Standardized % bias (unmatched)	Standardized % bias (matched)	% bias reduction
Age	-11.0	-0.9	91.8
Female sex	4.9	-0.7	85.9
Race/ethnicity	-3.8	2.2	42.6
Hyperlipidemia	-18.1	-0.2	98.8
Hypertension	-20.0	-0.3	98.3
Heart failure	-13.8	0.8	94.2
Prior myocardial infarction	-10.5	0.4	96.1
Prior PCI	-9.4	0.7	92.4
Prior CABG	-1.9	0.4	76.4
Obesity	-1.2	3.5	—
Chronic kidney disease	-16.7	0.4	97.3
Smoking/tobacco use	4.9	1.6	67.4
COPD	-5.8	1.9	66.7
Obstructive sleep apnea	-6.2	2.4	61.1
Prior stroke	-10.9	0.8	93.1
Alcoholic liver disease	-2.0	0.3	86.5
Liver cirrhosis/fibrosis	-5.5	2.2	60.5
Toxic liver disease	-0.1	0.0	100.0
Diabetes mellitus	-15.5	0.9	94.3
Hypothyroidism	-5.9	1.5	74.9
Nutritional anemia	-20.8	-0.6	97.3
COVID-19	0.2	1.2	—
Hospital region	3.6	-0.1	96.2
Location/teaching status	-5.3	-2.9	44.4
Hospital bed size	-1.7	-1.7	—
Primary payer	12.2	2.4	80.2

Standardized % bias for each covariate before (unmatched) and after (matched) 1:1 nearest-neighbor propensity score matching (caliper 0.1, common support). Overall mean absolute bias decreased from 8.2% to 1.2%, and Rubin's B from 38.0 to 7.9, indicating excellent post-match balance. Dashes denote covariates with negligible baseline imbalance for which percent reduction is not meaningfully defined.

Supplemental Table S4. Variance inflation factors for the multivariable logistic regression models.

Covariate	VIF	Tolerance (1/VIF)
Age 45–64	2.61	0.38
Age ≥65	3.59	0.28
Female sex	1.08	0.93
Race: Black	1.09	0.92
Race: Hispanic	1.08	0.92
Race: Asian/Pacific Islander	1.05	0.95
Race: Native American	1.01	0.99
Race: Other	1.02	0.98
Hyperlipidemia	1.27	0.79
Hypertension	1.36	0.73
Heart failure	1.16	0.86
Prior myocardial infarction	1.12	0.89
Prior PCI	1.12	0.89
Prior CABG	1.01	0.99
Obesity	1.11	0.90
Chronic kidney disease	1.18	0.85
Smoking/tobacco use	1.11	0.90
COPD	1.12	0.89
Obstructive sleep apnea	1.08	0.92
Prior stroke	1.04	0.96
Alcoholic liver disease	1.00	1.00
Liver cirrhosis/fibrosis	1.01	0.99
Toxic liver disease	1.00	1.00
Diabetes mellitus	1.18	0.85
Hypothyroidism	1.06	0.94
Nutritional anemia	1.01	0.99
COVID-19	1.00	1.00
Hospital region	1.05	0.96
Location/teaching status	1.04	0.96
Hospital bed size	1.02	0.98
Primary payer	1.48	0.68
**Mean VIF**	**1.23**	

Variance inflation factors (VIF) from the multivariable model. All VIF values were well below the conventional threshold of concern (VIF < 5), with a mean VIF of 1.23, indicating no meaningful multicollinearity among predictors. VIF values were identical across outcome models as they depend only on the predictor set.

Supplemental Table S5. Complete multivariable logistic regression results for all five outcomes (in-hospital mortality, sepsis, septic shock, acute kidney injury, and other/unspecified shock).

Predictor	Mortality aOR (95% CI)	Sepsis aOR (95% CI)	Septic shock aOR (95% CI)	AKI aOR (95% CI)	Other shock aOR (95% CI)
Age 45–64	2.80 (2.26–3.48)	1.52 (1.39–1.66)	2.22 (1.96–2.53)	2.01 (1.83–2.22)	1.26 (1.03–1.55)
Age ≥65	5.79 (4.60–7.28)	1.73 (1.57–1.91)	2.43 (2.11–2.79)	2.84 (2.56–3.14)	1.37 (1.10–1.72)
Female sex	0.91 (0.84–0.99)	0.92 (0.87–0.96)	0.82 (0.77–0.88)	0.71 (0.67–0.74)	0.70 (0.63–0.79)
Black	0.80 (0.70–0.91)	0.89 (0.83–0.96)	0.86 (0.78–0.95)	1.12 (1.04–1.20)	1.35 (1.15–1.57)
Hispanic	0.95 (0.82–1.10)	0.96 (0.88–1.05)	0.89 (0.79–1.00)	0.93 (0.85–1.01)	1.15 (0.94–1.40)
Asian/Pacific Islander	0.86 (0.69–1.07)	0.88 (0.76–1.00)	0.79 (0.66–0.93)	0.81 (0.72–0.91)	1.70 (1.35–2.15)
Native American	1.21 (0.77–1.90)	0.98 (0.74–1.30)	1.36 (0.99–1.86)	0.86 (0.63–1.18)	1.04 (0.53–2.05)
Other race	0.94 (0.73–1.21)	0.95 (0.82–1.10)	0.89 (0.74–1.08)	1.04 (0.89–1.20)	1.18 (0.85–1.64)
Hyperlipidemia	0.65 (0.59–0.72)	0.73 (0.69–0.77)	0.66 (0.61–0.71)	0.83 (0.79–0.88)	1.04 (0.91–1.18)
Hypertension	0.77 (0.70–0.84)	0.88 (0.83–0.93)	0.73 (0.68–0.78)	1.08 (1.02–1.14)	0.85 (0.74–0.97)
Heart failure	1.73 (1.56–1.92)	1.51 (1.41–1.62)	1.82 (1.67–1.98)	1.65 (1.55–1.76)	1.45 (1.26–1.68)
Prior MI	0.83 (0.68–1.03)	0.77 (0.67–0.88)	0.72 (0.61–0.86)	0.81 (0.72–0.91)	0.97 (0.75–1.27)
Prior PCI	0.54 (0.42–0.69)	0.49 (0.42–0.57)	0.42 (0.34–0.52)	0.73 (0.65–0.82)	0.97 (0.75–1.27)
Prior CABG	0.93 (0.43–2.02)	0.73 (0.40–1.31)	0.63 (0.30–1.32)	0.82 (0.49–1.36)	2.29 (1.05–5.01)
Obesity	0.99 (0.89–1.11)	1.28 (1.19–1.37)	1.36 (1.26–1.48)	1.34 (1.25–1.43)	1.09 (0.94–1.27)
Chronic kidney disease	1.41 (1.27–1.56)	1.14 (1.06–1.22)	1.33 (1.22–1.45)	2.81 (2.64–2.98)	1.19 (1.03–1.37)
Smoking/tobacco use	0.57 (0.52–0.62)	0.74 (0.70–0.78)	0.55 (0.51–0.59)	0.72 (0.69–0.76)	0.73 (0.65–0.82)
COPD	1.55 (1.40–1.72)	1.53 (1.43–1.63)	1.59 (1.47–1.72)	1.20 (1.13–1.28)	1.11 (0.96–1.29)
Obstructive sleep apnea	0.73 (0.60–0.88)	0.74 (0.66–0.83)	0.69 (0.59–0.80)	0.72 (0.65–0.80)	0.84 (0.65–1.08)
Prior stroke	0.83 (0.71–0.97)	0.76 (0.69–0.84)	0.77 (0.68–0.88)	0.85 (0.78–0.94)	1.06 (0.86–1.30)
Alcoholic liver disease	0.47 (0.17–1.31)	0.66 (0.41–1.08)	0.69 (0.37–1.31)	0.94 (0.63–1.42)	1.17 (0.46–2.95)
Liver cirrhosis/fibrosis	1.93 (1.62–2.30)	1.30 (1.14–1.48)	1.38 (1.19–1.61)	1.33 (1.18–1.50)	1.82 (1.43–2.31)
Toxic liver disease[1]	2.11 (0.16–27.70)	—	—	2.88 (0.72–11.60)	—
Diabetes mellitus	1.04 (0.95–1.15)	1.13 (1.07–1.21)	1.12 (1.04–1.21)	1.19 (1.12–1.26)	1.04 (0.91–1.19)
Hypothyroidism	0.78 (0.68–0.89)	0.83 (0.77–0.91)	0.76 (0.68–0.84)	0.89 (0.83–0.96)	0.98 (0.81–1.18)
Nutritional anemia	0.67 (0.57–0.78)	0.74 (0.68–0.82)	0.67 (0.60–0.76)	0.98 (0.90–1.06)	0.68 (0.54–0.86)
COVID-19	4.35 (3.46–5.47)	2.35 (1.93–2.86)	2.37 (1.91–2.95)	2.39 (1.96–2.91)	2.56 (1.86–3.52)
Hospital region	1.00 (0.96–1.05)	1.08 (1.05–1.11)	1.06 (1.02–1.09)	1.04 (1.01–1.06)	1.01 (0.96–1.07)

Predictor	Mortality aOR (95% CI)	Sepsis aOR (95% CI)	Septic shock aOR (95% CI)	AKI aOR (95% CI)	Other shock aOR (95% CI)
Location/teaching status	1.29 (1.21–1.38)	1.19 (1.14–1.24)	1.39 (1.32–1.46)	1.24 (1.20–1.29)	1.52 (1.37–1.68)
Hospital bed size	1.19 (1.13–1.26)	1.09 (1.06–1.13)	1.22 (1.17–1.27)	1.08 (1.05–1.12)	1.28 (1.19–1.38)
Primary payer	1.04 (0.99–1.08)	0.95 (0.93–0.98)	0.91 (0.88–0.94)	0.95 (0.93–0.98)	0.92 (0.87–0.98)

aOR = adjusted odds ratio; CI = confidence interval; MI = myocardial infarction; PCI = percutaneous coronary intervention; CABG = coronary artery bypass grafting; COPD = chronic obstructive pulmonary disease; AKI = acute kidney injury. All models adjusted for the covariates listed plus hospital-level characteristics. [1]Toxic liver disease estimates are based on very small cell counts (n=35) and are statistically unstable; the variable was omitted from the sepsis, septic shock, and other/unspecified shock models due to perfect prediction. Reference groups: age 18–44, White race.
