# Supplementary material for: Outcomes and Predictors of Mortality in Perforated Versus Non-Perforated Peptic Ulcer Disease: A U.S. Nationwide Propensity-Matched Analysis, 2016–2021
Source: J Clin Med. 2026 Jun 4;15(11):4358. doi: 10.3390/jcm15114358 (PMC13257457; doi:10.3390/jcm15114358)
Supplement: Supplementary file 1 [file jcm-15-04358-s001.zip › Supplemental Table S2.pdf]

Supplemental Table S2. Covariates included in the propensity score model and the multivariable regression models.

Category	Variable
**Demographics**	Age
	Sex
	Race/Ethnicity
**Cardiovascular conditions**	Hyperlipidemia
	Hypertension
	Heart failure
	Prior myocardial infarction
	Prior percutaneous coronary intervention
	Prior coronary artery bypass grafting
	Prior stroke
**Endocrine and metabolic conditions**	Obesity
	Diabetes mellitus
	Hypothyroidism
**Pulmonary conditions**	Chronic obstructive pulmonary disease
	Obstructive sleep apnea
	COVID-19 infection
**Renal conditions**	Chronic kidney disease / End-stage renal disease
**Liver conditions**	Alcoholic liver disease
	Toxic liver disease
	Liver cirrhosis / fibrosis
**Other**	Smoker / tobacco user
	Nutritional anemia

Footnote: *All variables listed above were included as covariates in the multivariable logistic regression model used to estimate propensity scores for 1:1 nearest neighbor matching without replacement. The same set of variables was entered into the multivariable logistic regression analyses performed within the perforated peptic ulcer disease cohort to identify independent predictors of in-hospital mortality, sepsis, septic shock, acute kidney injury, and other/unspecified shock. ICD-10-CM codes used to define each comorbidity are provided in Supplemental Table S1.*
