# Supplementary figures and images for: Outcomes and Predictors of Mortality in Perforated Versus Non-Perforated Peptic Ulcer Disease: A U.S. Nationwide Propensity-Matched Analysis, 2016–2021
Source: J Clin Med. 2026 Jun 4;15(11):4358. doi: 10.3390/jcm15114358 (PMC13257457; doi:10.3390/jcm15114358)

# Balance plot

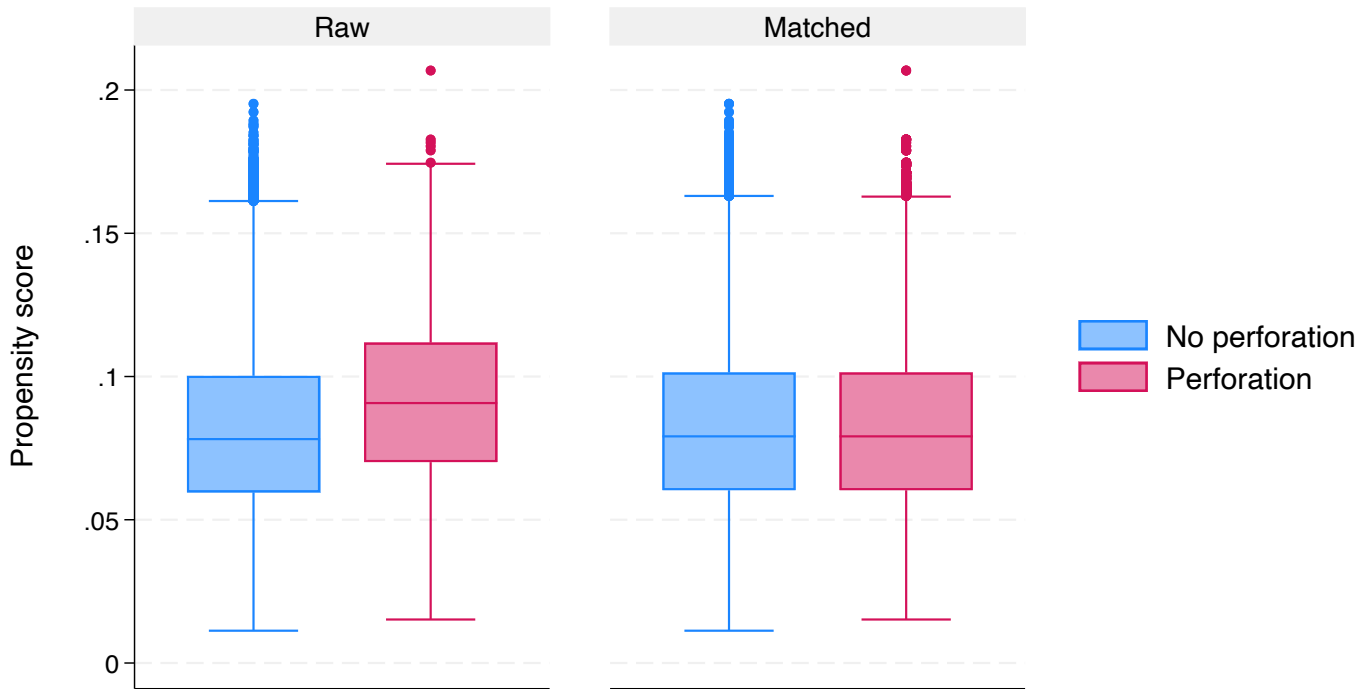

Supplement: Supplementary file 1 [file jcm-15-04358-s001.zip › Supplemental Figure S1.pdf]
